# Altered Endothelin Receptor Expression and Affinity in Spontaneously Hypertensive Rat Cerebral and Coronary Arteries

**DOI:** 10.1371/journal.pone.0073761

**Published:** 2013-09-02

**Authors:** Lei Cao, Yong-Xiao Cao, Cang-Bao Xu, Lars Edvinsson

**Affiliations:** 1 Division of Experimental Vascular Research, Institute of Clinical Sciences in Lund, Lund University, Sweden; 2 Department of Pharmacology, Xi’an Jiaotong University College of Medicine, Xi’an, Shaanxi, People’s Republic of China; 3 Institute of Basic and Translational Medicine, Xi’an Medical University, Xi’an, Shaanxi, People’s Republic of China; University of Pécs Medical School, Hungary

## Abstract

**Background:**

Hypertension is associated with arterial hyperreactivity, and endothelin (ET) receptors are involved in vascular pathogenesis. The present study was performed to examine the hypothesis that ET receptors were altered in cerebral and coronary arteries of spontaneously hypertensive rats (SHR).

**Methodology/Principal Findings:**

Cerebral and coronary arteries were removed from SHR. Vascular contraction was recorded using a sensitive myograph system. Real-time PCR and Western blotting were used to quantify mRNA and protein expression of receptors and essential MAPK pathway molecules. The results demonstrated that both ET_A_ and ET_B_ receptor-mediated contractile responses in SHR cerebral arteries were shifted to the left in a nonparallel manner with increased maximum contraction compared with Wistar-Kyoto (WKY) rats. In SHR coronary arteries, the ET_A_ receptor-mediated contraction curve was shifted to the left in parallel with an increased pEC_50_ compared with the arteries in WKY rats. There was no significant increase in ET_B_ receptor-mediated contraction in SHR coronary arteries. ET_A_ receptor mRNA and protein expression was increased in SHR cerebral arteries compared with the arteries in WKY rats. However, ET_A_ receptor mRNA and protein levels in coronary arteries and ET_B_ receptor protein levels in cerebral and coronary arteries remained unchanged in SHR compared with WKY rats. Meanwhile, phosphorylated ERK1/2 protein was significantly increased in SHR brain and heart vessels.

**Conclusions/Significance:**

In SHR cerebral arteries, ET_A_ receptor expression was upregulated. ET_A_ receptor affinity was increased in coronary arteries, and ET_B_ receptor affinity was increased in cerebral arteries. The ERK1/2 activation may be involved in the receptor alterations.

## Introduction

Vascular smooth muscle cell receptors mediate vasoconstriction and vasodilatation, which are of key importance in vascular resistance and blood pressure (BP) regulation. Receptor metabolism updates constantly and is kept in a dynamic balance. Receptor regulation is an important factor for maintaining organismal homeostasis by changing receptor density and/or affinity and target cell sensitivity [1]. Numerous studies have demonstrated that receptor regulation plays a prominent role among adaptive changes in the cardiovascular system under pathological conditions [2]. Therefore, altered receptor expression is important for regulating physiological function, which may contribute to diverse pathological processes and could be involved in vascular pathologies such as hypertension.

Hypertension is a major risk factor for many cardiovascular events [3]. There is a clear correlation between increased BP and cardiovascular risk because increased BP increases the risk of stroke, myocardial infarct and heart failure [4]. Pathological arteriole processes are the most significant features of hypertension. The endothelin (ET) system consisting of ligands and their receptors is an important modulator of vascular tone and blood pressure, which play an essential role in cardiovascular pathogenesis [5]. Endothelin-1 (ET-1) is a potent vasoconstrictor that is formed in endothelial cells and participates in vascular regulation such as controlling basal arterial tone, altering vessel diameter and modifying blood flow [6]. ET-1 induces its vasoactive response through two distinct transmembrane receptors: endothelin type A (ET_A_) and endothelin type B (ET_B_) [7,8]. The ET_A_ receptor mediates vasoconstriction and vascular smooth muscle cell proliferation, whereas the ET_B_ receptor is involved in endothelial cell survival, ET reuptake, clearance, nitric oxide, and prostacyclin release. The ET_A_ receptor is expressed in the vascular smooth muscle of most blood vessels [9–11]. While the ET_B_ receptor predominates in endothelial cells, it is also present in the vascular smooth muscle of some vascular beds [12,13]. The prominent and long-acting vasoconstrictor effects of ET-1 may play a role in blood pressure regulation and hypertension pathophysiology [14]. Upregulation of the ET system is more commonly observed in severe hypertension and is associated with coronary artery disease, heart failure, and atherosclerosis. ET-1 plasma levels increase in some hypertensive patients. ET-1 mRNA expression is increased in the endothelium of subcutaneous resistance arteries from patients with moderate to severe hypertension [13].

Variability in the ET system in cardiovascular pathogenesis may not only involve ET-1 levels, but could also involve the amount of vascular ET receptors. Increased ET_A_/ET_B_ receptor expression has been reported in blood vessels of cardiovascular disease patients [15–17]. Vascular ET receptor numbers could vary for different forms of hypertension and in various tissues isolated from subjects with the same form of hypertension. In a previous study, we reported that ET_B_ and ET_A_ receptor-mediated contractions were increased in the mesenteric artery of spontaneously hypertensive rats (SHR) and the colon marginal artery of hypertension patients [8]. Studies have demonstrated that ET-1-induced contraction is increased in rat heart coronaries during ischemia/reperfusion [18]. ET_B_ receptors are upregulated in pulmonary hypertension [19]. ET receptors could also be downregulated when large amounts of ET are produced in the vasculature [9]. In addition, risk factors for vascular diseases, such as low-density lipoprotein and smoking, induce receptor upregulation in different vessels [20,21]. ET receptor antagonists reduce BP to a variable extent in hypertension [22], supporting the notion that the ET system is upregulated in hypertension. Therefore, we hypothesized that arterial ET receptor alterations may occur in hypertension.

In the present study, we focused on two key regions, the cerebral and coronary arteries. In SHR, we examined whether basal ET receptor levels were altered in cerebral and coronary arteries. Receptor-mediated vascular contractile responses together with receptor mRNA and protein expression levels were studied. We also addressed the possible mechanism behind the receptor modification by assessing the phosphorylation of the three main mitogen-activated protein kinase (MAPK) signaling pathways: extracellular signal-regulated kinase (ERK) 1/2, c-Jun N-terminal kinase (JNK) and p38.

## Materials and Methods

### Animals

In total, 20 male SHR and 24 Wistar-Kyoto (WKY) rats weighing 250-300 g (20 weeks) were obtained from Slac Laboratory Animal Co., Ltd. (Shanghai, China). The rats were acclimated for one week under standardized temperature (21-22°C) and humidity (50-60%) with free access to food and water before the experiment. All experimental protocols were approved by the Xi’an Jiaotong University Animal Ethics Committee.

### BP Measurement

Arterial pressure was measured in SHR and WKY *via* a non-invasive tail-cuff plethysmography method [23] and monitored with the CODA 6 Non-Invasive Blood Pressure System (Kent Scientific, Torrington, CT, USA) before euthanasia. Systolic and diastolic tail arterial BP were 218 ± 15/173±9 mm Hg in SHR and 110 ± 5/70±4 mm Hg in the WKY rats. The recorded BP was an average of three readings.

### Artery Collection

Rats were anesthetized with CO_2_ and decapitated. The brains and hearts were immediately removed and immersed in an ice-cold bicarbonate buffer solution [24]. The basilar artery, middle cerebral artery and circle of Willis artery were carefully removed from the brain. The left anterior descending (LAD) coronary artery, left circumflex artery and right coronary artery were isolated from the heart. Some basilar arteries and LAD coronary arteries were cut into cylindrical segments (1-2 mm in length) for *in vitro* pharmacology studies. Several of the remaining basilar arteries and LAD coronary arteries were frozen with other isolated arteries at -80°C for real-time PCR and Western blotting.

### Vascular Ring Myograph Studies

Sensitive myographs (Danish Myo Technology A/S, Aarhus, Denmark) were used for recording isometric tension of isolated arteries [25]. The vessels were cut into cylindrical segments, threaded onto two 40 µm diameter stainless steel wires and mounted in the myograph chamber. One wire was connected to a force displacement transducer that was attached to a digital converter unit. Another wire was connected to a micrometer screw, which allowed for fine vascular tone adjustments by varying the distance between the wires. Measurements were recorded in a computer using a PowerLab Unit (ADInstruments, Oxford, UK). The segments were immersed in a temperature-controlled buffer solution (37°C), which was continuously equilibrated with a 5% CO_2_ in O_2_ gas mixture resulting in a stable pH of 7.4. The vessels were given an initial tension of 1-1.2 mN and were adjusted to this level of tension for at least 1 h. Potassium-rich (60 mM) buffer solution was used to determine segment contractile function as a contractile capacity reference.

The concentration–response curves of vascular segment were obtained by cumulative ET_B_ receptor agonist sarafotoxin 6c (S6c, 10^-11^-10^-7^ M, Merck, Darmstadt, Germany) administration and both ET_A_ and ET_B_ receptor agonist ET-1 (10^-11^-10^-7^ M, NeoMPS, Strasbourg, France) administration. To evaluate ET_A_ receptor-mediated contraction, a concentration-contractile response to S6c (10^-11^-10^-7^ M) was performed in advance to achieve ET_B_ receptor desensitization [26]. When a maximal contraction (E_max_) induced by S6c was reached, the segments remained in contact with the highest S6c concentration for 30 additional min until the contractile curves faded to baseline level, which was considered to be total desensitization. Thus, ET-1 (10^-11^-10^-7^ M) induced a concentration-effect curve, which was only mediated by ET_A_ receptors [27].

### Real-time PCR

Cerebral and coronary arteries were collected as described above. Total cellular RNA was extracted using the RNeasy Mini Kit (Qiagen GmbH, Hilden, Germany) following the supplier’s instructions. RNA purity was assessed with an Eppendorf Biophotometer (Hamburg, Germany), and the wavelength/absorption ratio (260/280 nm) ranged from 1.7 to 2.0 for all of the preparations. Reverse transcription of total RNA to cDNA were performed in 40 µl reaction volume using TaqMan Reverse Transcription Reagents (Applied Biosystems, Foster City, CA, USA) in a PerkinElmer 2400 PCR machine (PerkinElmer, Waltham, MA, USA). Real-time PCR was performed using the GeneAmp SYBR^®^ Green kit (Applied Biosystems) in a GeneAmp 7300 sequence Detection System (Applied Biosystems). Details were previously described [28]. Specific primers for the receptors were designed as follows: ET_A_ receptor forward: 5’-GTC GAG AGG TGG CAA AGA CC-3’, reverse: 5’-ACA GGG CGA AGA TGA CAA CC-3’ and ET_B_ receptor forward: 5’-GAT ACG ACA ACT TCC GCT CCA-3’, reverse: 5’-GTC CAC GAT GAG GAC AAT GAG-3’. Elongation factor-1 (EF-1) was used as an endogenous reference standard. EF-1 primers were forward: 5’-GCA AGC CCA TGT GTG TTG AA-3’ and reverse: 5’-TGA TGA CAC CCA CAG CAA CTG -3’. The data were analyzed using the comparative cycle threshold (C_T_) method [29].

### Western Blotting

Cerebral and coronary arteries were harvested as mentioned above. Vessel proteins were extracted as described previously [30]. Equal amounts of protein (40 µg) were loaded onto a 4-15% Ready Gel Precast Gel (Bio-Rad Laboratories, Hercules, CA, USA) for electrophoresis. A molecular weight marker was loaded for protein band identification. Proteins were then transferred to a nitrocellulose membrane (Bio-Rad Laboratories). The membrane was blocked in 5% non-fat milk and incubated with primary antibodies at 4°C overnight. Primary antibodies were as follows: 1:200 rabbit anti-ET_A_ receptor (sc-33535, Santa Cruz Biotechnology, CA, USA), 1:500 rabbit anti-ET_B_ receptor (ab65972, Abcam, Cambridge, UK), 1:2000 rabbit anti-phospho-ERK1/2 (#4370, Cell Signaling Technology, Beverly, MA), 1:1000 rabbit anti-phospho-SAPK/JNK (#4668, Cell Signaling Technology), 1:1000 rabbit anti-phospho-p38 (#4631, Cell Signaling Technology), 1:1000 mouse anti-β-actin (#4970, Cell Signaling Technology) or 1:2000 mouse anti-ERK1/2 (#4696, Cell Signaling Technology). Next, membranes were incubated with 1:2000 HRP-conjugated anti-rabbit (#7074, Cell Signaling Technology) or anti-mouse (#7076, Cell Signaling Technology) secondary antibodies for 1 h at room temperature. Finally, membranes were developed and visualized using a Fujifilm LAS-1000 Luminescent Image Analyzer (Fujifilm, Stamford, CT, USA). Band intensity was quantified using ImageJ software (http://rsb.info.nih.gov/ij/).

### Statistical Analysis

All of the data are expressed as the mean ± SEM, and *n* refers to the number of rats. Contractile responses to receptor agonists in each segment were expressed as a percentage of the contraction that was induced by 60 mM K^+^-rich buffer solution. Target gene mRNA levels were expressed in relation to EF-1 levels. Target protein expression was determined relative to β-actin levels or total ERK1/2 protein levels. Unpaired Student’s *t*-test was used to compare two data sets. 2-way ANOVAs were used to compare two corresponding data points at each concentration on the two curves. The data and statistical analysis was calculated using Graph-Pad Prism 5.0 (GraphPad Software, La Jolla, CA, USA). *P* < 0.05 was considered to be statistically significant.

## Results

### ET Receptor-Mediated Contractile Responses in Cerebral and Coronary Arteries

Cerebral basilar arteries and LAD coronary arteries were examined separately. K^+^-induced basilar artery or coronary artery contractile responses in SHR did not differ significantly from the responses in WKY (Table 1). However, K^+^-induced contraction on basilar arteries was stronger than in coronary arteries from SHR. Because KCl is a receptor-independent agonist, receptor agonist-mediated contractions relative to KCl-induced contractions (as a percentage of the KCl-mediated contraction) provided an indication of agonist sensitivity. Therefore, the K^+^-elicited contraction was used as a contractile capacity reference in each specific vessel.

**Table 1:** E_max_ and pEC_50_ values of the basilar and coronary arterial segment concentration-contractile curves isolated from endothelin receptor agonist-induced spontaneously hypertensive rats.

Group (*n*)		S6c		ET-1	
		WKY (14)	SHR (10)	WKY (14)	SHR (10)
Basilar	K^+^ (mN)	2.53±0.27	3.27±0.33	2.55±0.26	3.33±0.31
	E_max_ (% of K^+^)	5.41±2.16	17.1±5.10^*^	112±3.67	150±7.85^**^
	pEC_50_	8.18±0.58	8.31±0.63	8.96±0.06	8.92±0.10
Coronary	K^+^ (mN)	1.84±0.22	1.61±0.28	1.98±0.18	1.72±0.24
	E_max_ (% of K^+^)	4.85±3.18	11.8±3.70	156±5.78	152±5.00
	pEC_50_	7.41±0.46	8.37±0.50	8.31±0.05	8.75±0.05^**^

Dates are expressed as the means±SEM, and *n* refers to the rat number. S6c: Sarafotoxin 6c; ET-1: endothelin-1; E_max:_ maximal contraction; pEC_50_: negative logarithm of the agonist concentration that produces 50% of E_max_. E_max_ are expressed as a percentage of 60 mM K^+^-induced contraction. ^*^ *P*<0.05, ^**^ *P*<0.01 *vs.* WKY group.

**Figure 1:**
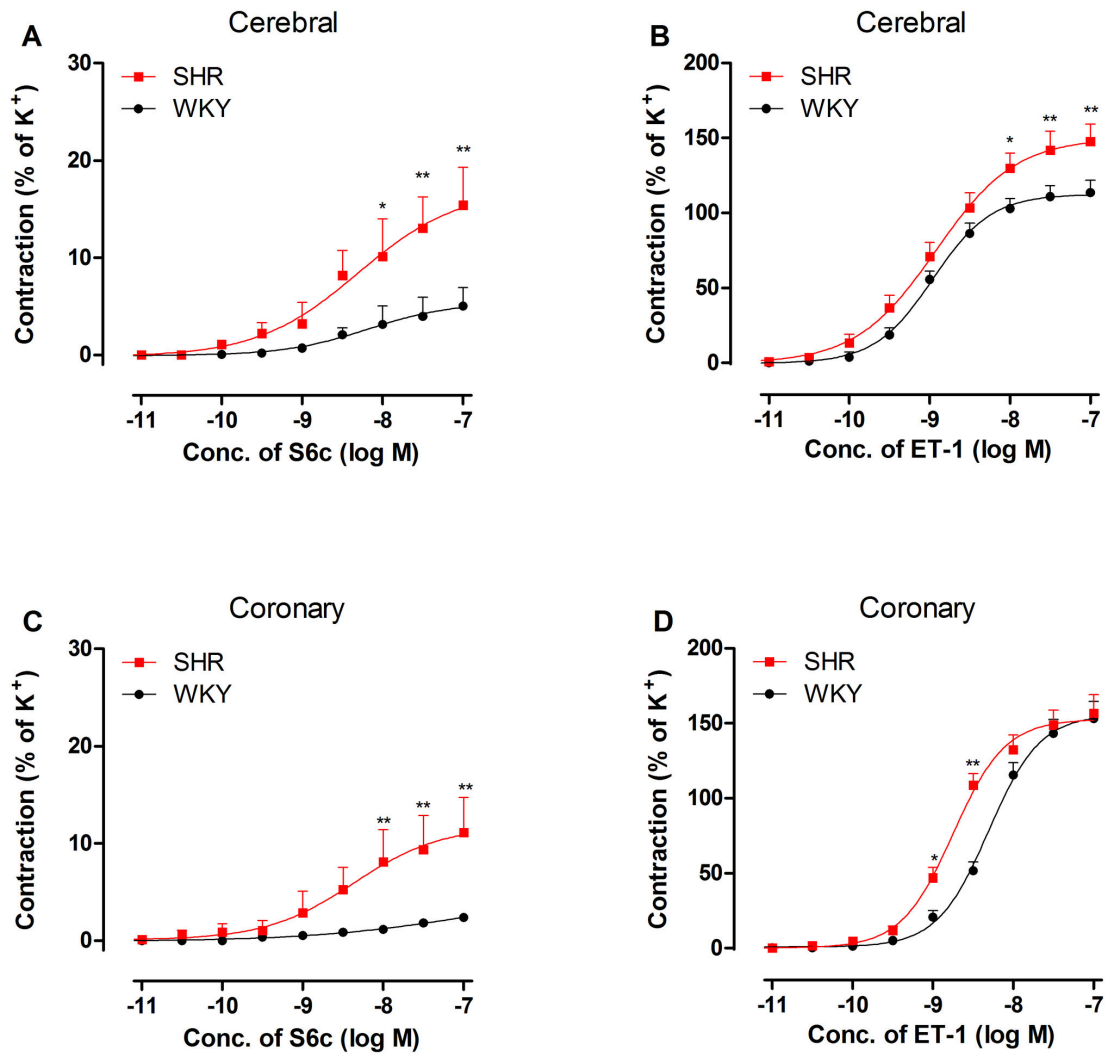
Concentration–response curves for cumulative sarafotoxin 6c (S6c, A and C) and endothelin-1 (ET-1, B and D) application in SHR and WKY cerebral (A, B) and coronary (C, D) arteries. Contractile responses were demonstrated as the percentage of K^+^-induced contraction. Each data point is derived from 10–14 animals and is expressed as the mean ± SEM. Statistical analysis was performed using a two-way ANOVA. ^*^ *P*<0.05, ^**^ *P*<0.01 *vs*. WKY group.

The selective ET_B_ receptor agonist S6c induced weak contractile responses in WKY basilar (Figure 1A) and coronary arteries (Figure 1C). E_max_ values were approximately 5% of K^+^-induced contractions. Figure 1 demonstrates that S6c induced a significantly stronger contractile response in SHR (E_max_ 17.10 ± 5.10%) compared with WKY (E_max_ of 5.41±2.16%, *P*<0.05) in cerebral arteries. However, S6c-induced maximal contraction was not significantly increased in SHR coronary arteries compared with WKY (11.80 ±3.70% *vs.* 4.85±3.18%, *P*>0.05). Additionally, there was no statistical difference of pEC_50_ values in the cerebral or coronary artery between WKY and SHR (Table 1).

ET-1, which activates both ET_B_ and ET_A_ receptors, was applied to activate only ET_A_ receptors following a total ET_B_ receptor desensitization. Cumulative ET-1 administration induced potent contractions in basilar (Figure 1B) and coronary (Figure 1D) arteries. Basilar artery contractile responses were stronger in SHR than in WKY (Figure 1B) with a significantly elevated E_max_ (150 ± 7.85% *vs.* 112 ± 3.67%, *P* < 0.01). In contrast, the ET_A_ receptor-mediated coronary artery contractile response curve in SHR was shifted toward the left in parallel with an increased pEC_50_ and an unchanged E_max_ (Figure 1D, Table 1).

### ET Receptor mRNA and Protein Expression in Cerebral and Coronary Arteries

**Figure 2:**
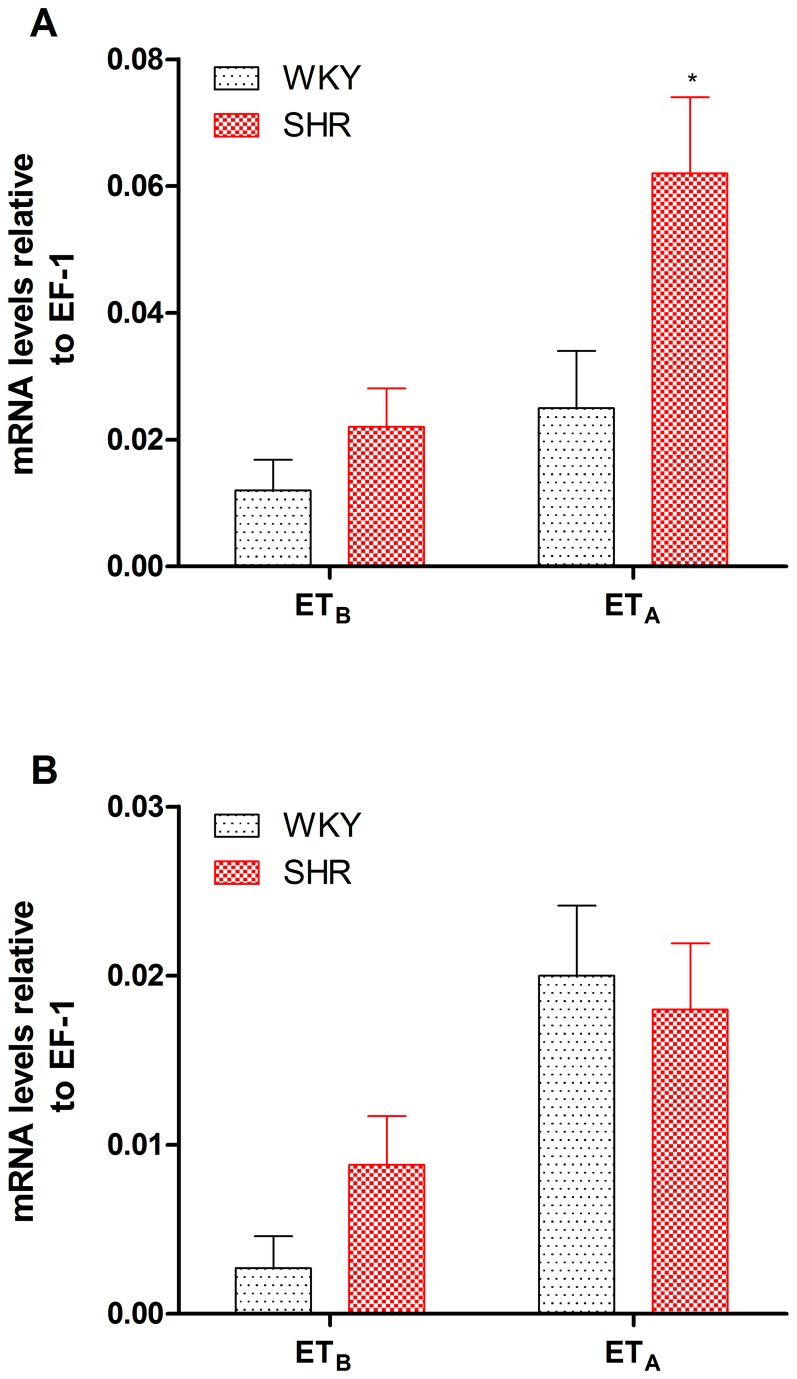
ET_B_ and ET_A_ receptor mRNA expression levels in SHR and WKY cerebral (A) and coronary (B) arteries. ET_B_ and ET_A_ receptor mRNA levels were expressed relative to elongation factor-1 (EF-1) levels. Each data point is derived from 7–8 animals, and the data are expressed as the mean ± SEM. Statistical analysis was performed using an unpaired Student’s *t*-test. ^*^ *P*<0.05 *vs*. WKY group.

Relative ET_B_ and ET_A_ receptor mRNA levels were quantified by real-time PCR. ET_A_ receptor mRNA levels were significantly increased (Figure 2A) in SHR basilar segments. It appeared that ET_B_ receptor mRNA levels tended to increase, but this was not statistically significant (Figure 2A, P > 0.05). Figure 2B demonstrates that in SHR coronary arteries, ET_A_ receptor mRNA was not altered compared with WKY. Although there was a trend toward increased ET_B_ receptor mRNA expression, there were no statistically significant differences between SHR and WKY (*P*<0.05, Figure 2B).

ET_B_ and ET_A_ receptor protein expression was examined by Western blotting.

**Figure 3:**
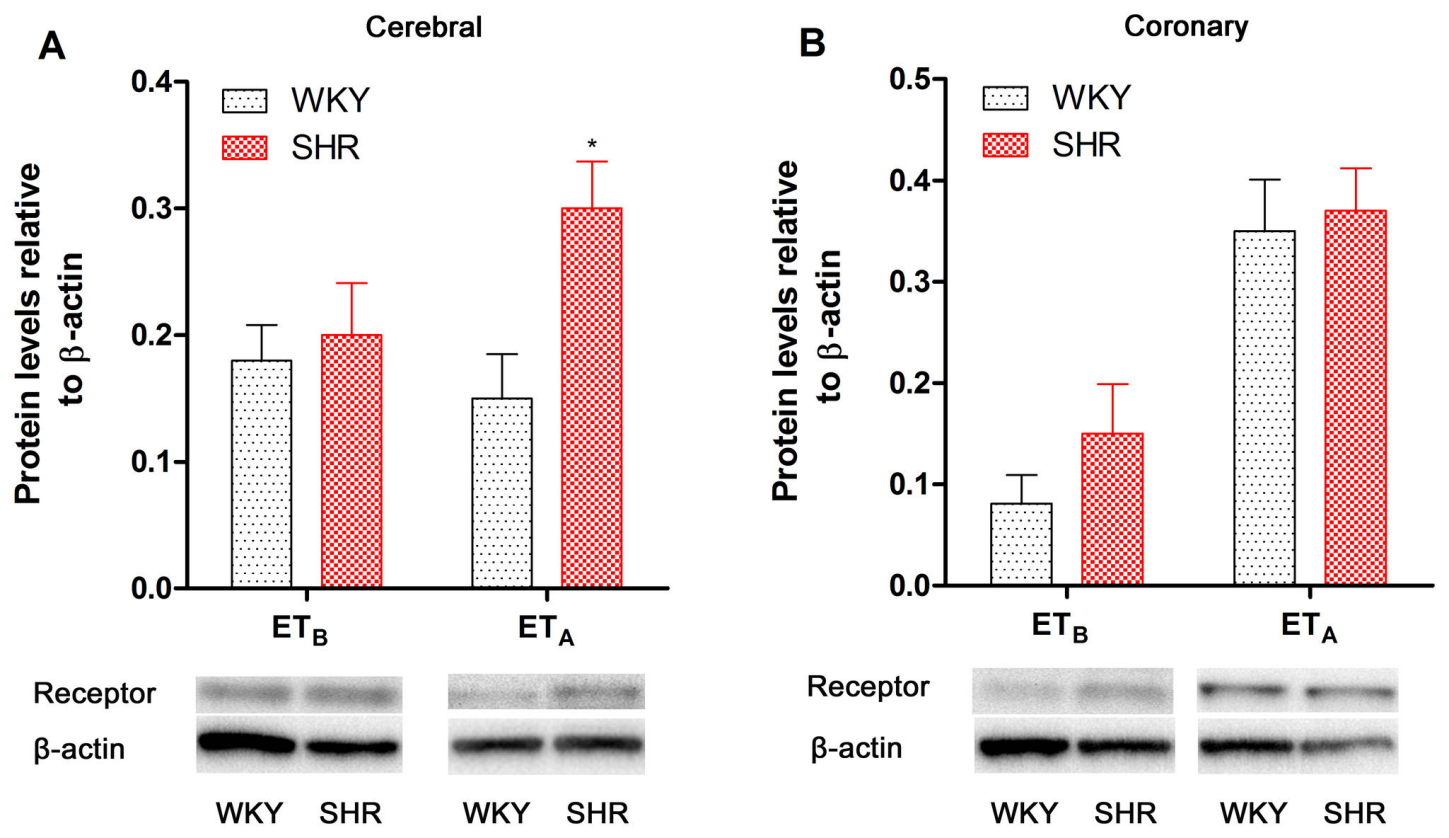
ET_B_ and ET_A_ receptor protein expression levels in SHR and WKY rat cerebral (A) and coronary (B) arteries. ET_B_ and ET_A_ receptor protein expression levels were expressed relative to β-actin levels. Each data point is derived from 7–8 animals and is expressed as the mean ± SEM. Statistical analysis was performed using an unpaired Student’s *t*-test. ^*^ *P*<0.05 *vs*. WKY group.

The results from cerebral artery segments demonstrated that ET_A_ receptor expression was increased while ET_B_ receptor expression was unaltered in SHR compared with WKY (Figure 3A). There was no significant change in ET_B_ or ET_A_ receptor protein expression in SHR coronary arteries (Figure 3B).

### MAPK Activities in Cerebral and Coronary Arteries

**Figure 4:**
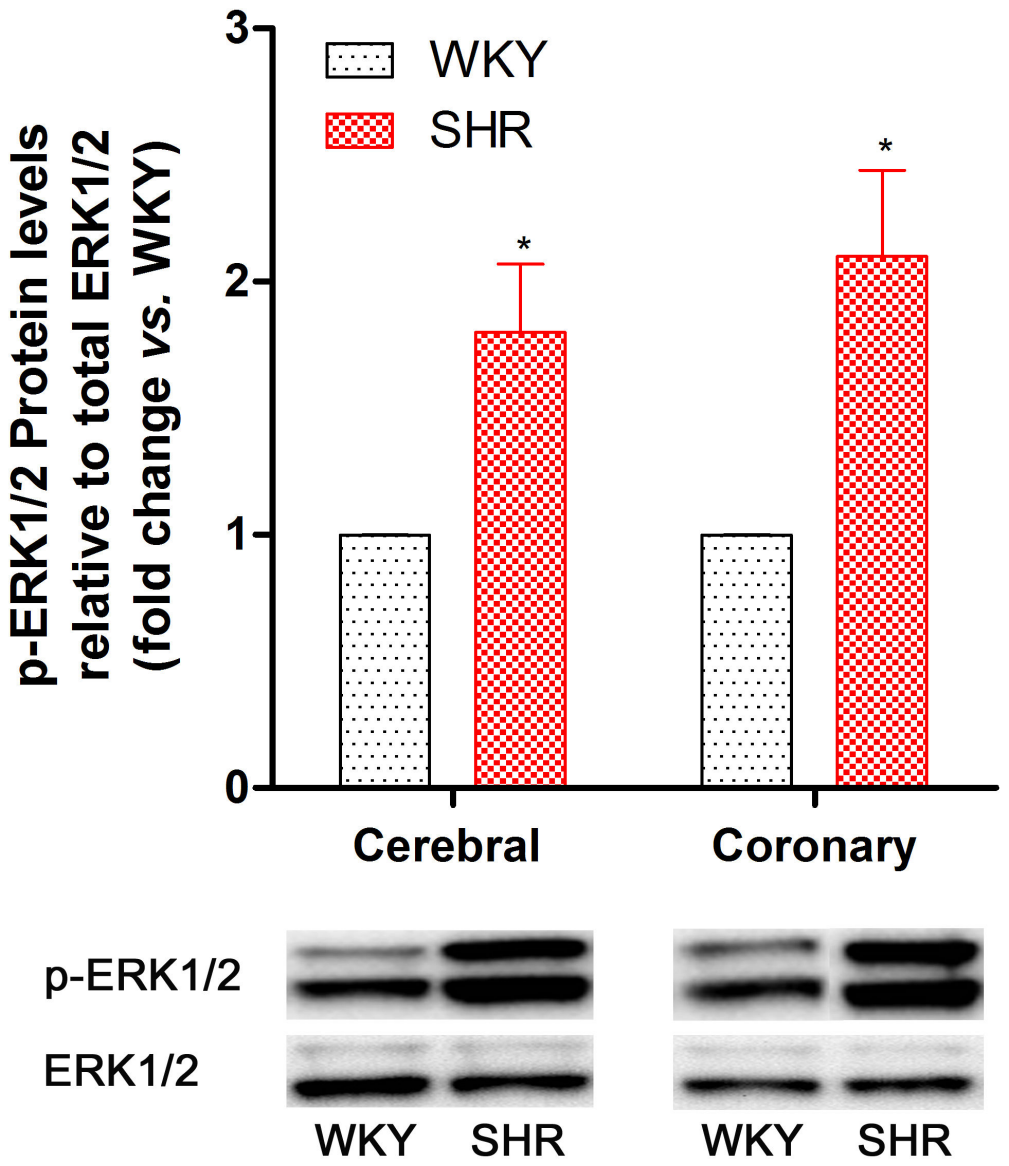
ERK1/2 protein phosphorylation in rat cerebral and coronary arteries. Phosphorylated (p)-ERK1/2 levels in SHR group relative to the WKY group. Each data point is derived from 7–8 animals, and the data are expressed as the mean ± SEM. Statistical analysis was performed using an unpaired Student’s *t*-test. ^*^ *P*<0.05 *vs*. WKY group.

To investigate the underlying intracellular signal transduction mechanisms involved in the vascular receptor alteration that occurred in SHR, MAPK activities were examined by Western blotting. We analyzed total and phosphorylated (p) ERK1/2, JNK and p38 MAPK protein expression to determine which pathways were activated during hypertension. The results demonstrated that p-ERK1/2 protein content was 1.8-fold higher in cerebral arteries and 2.1-fold higher in coronary arteries in SHR compared with vessels from WKY (Figure 4). However, neither p-JNK nor p-p38 proteins were altered in SHR and WKY vascular segments.

## Discussion

The main finding of the present study is that there is significantly increased cerebral ET_A_ receptor expression in SHR compared with WKY. ET_A_ receptor mRNA and protein expression was increased in cerebral vessels, which was reflected by enhanced ET-1-induced smooth muscle contraction. However, ET_B_ receptor expression was unaltered in cerebral arteries, and ET_B_ and ET_A_ receptor expression was unchanged in coronary arteries. Coronary artery ET_A_ receptor and cerebral artery ET_B_ receptor affinities were increased. ERK1/2 phosphorylation was also increased in both cerebral and coronary arteries.

Resistance in arteries and arterioles profoundly influences BP. Relatively small changes in blood vessel diameter may dramatically alter vascular resistance. Blood vessel radius is maintained by a delicate balance of vasoconstrictive and vasodilatory inputs. As a major contributor to hypertension development, the vascular smooth muscle contractile state regulates the vessel radius thus modulating peripheral resistance [31]. ET_B_ and ET_A_ receptor agonists can induce vasoconstriction or vasodilatation by binding to their respective receptors [9].

Lariviere et al. [32] studied ET-1 gene expression in blood vessels of adult SHR. Their results indicated reduced or normal vascular ET-1 content in the SHR aorta and mesenteric arteries. They concluded that ET-1 did not play an important role in the pathogenesis of elevated BP in the SHR model of genetic hypertension. Their results suggest that ET receptor density and/or activity might be upregulated because of reduced vascular ET-1 content, which is consistent with our present results.

Receptor agonist-induced concentration–response curves can be determined by two important parameters, E_max_ and EC_50_. E_max_ refers to receptor-mediated maximal response, which reflects receptor efficacy. Based on the receptor occupation theory, the receptor effect is proportional to receptor number. Thus, the E_max_ reflects receptor number. EC_50_ is the concentration of agonist that can produce a response equal to 50% of E_max_, which is used to determine receptor affinity or potency. The present study revealed that ET_A_ receptor-mediated contractile responses were enhanced in SHR cerebral arteries with an increased E_max_ and an unchanged pEC_50_ compared with WKY. These data suggest that the receptor number may be increased. Further results demonstrated that ET_A_ receptor mRNA and protein expression in the cerebral artery was elevated compared with WKY. The results are in concert with contractile function studies, indicating that the cerebral artery ET_A_ receptor is upregulated in SHR. Similar results have been documented in cerebral arteries of subarachnoid hemorrhage [33], demonstrating that the increased ET-1 sensitivity is caused by increasing smooth muscle receptor expression. In support of these data, ET_A_ receptor density is increased in cerebral arterioles of the stroke-prone SHR [34]. The enhanced vasoconstriction that was observed may be attributed to transcription and *de novo* contractile receptor translation [35].

Increased ET receptor expression has been found in coronary arteries of ischemic heart disease patients [17] and in cerebral artery of ischemic/hemorrhagic stroke patients [15,16], suggesting that ET receptor regulation is a novel and possibly important feature in many cardiovascular diseases [2]. Therefore, we speculated that ET receptor expression might be altered in SHR arteries. The present study focused on the cerebral and coronary arteries, which are in two of the most important organs. Vascular ET receptor number may vary in different organs [36]. The present study demonstrated that ET_A_ receptor-mediated coronary artery contractile response curves in SHR were shifted toward the left in parallel with an increased pEC_50_ and an unchanged E_max_, suggesting that ET_A_ receptor affinity increases, whereas the receptor number may not be increased. The myograph results were consistent with mRNA and protein levels. There were no significant differences in ET_A_ receptor mRNA and protein levels in coronary arteries of SHR compared with WKY. These data suggest that ET_A_ receptor number does not increase in SHR coronary arteries. Similar results appear in rat heart coronary arteries during ischemia/reperfusion [18]. The augmented vascular reactivity to ET-1 in experimental hypertension may be related to increased intracellular free Ca^2+^ concentration in vascular smooth muscles [37,38].

The ET_B_ receptor agonist S6c-induced cerebral and coronary artery contractions were weak, and the mRNA and protein expression levels were low in WKY, which can be ignored. There was no direct functional response to the selective S6c-mediated ET_B_ stimulation. Beg et al. described a dimerization mechanism between the two receptors to form an ET_A_-ET_B_ receptor heterodimer in ET-1 recognition that represents a functional ET_B_ receptor [39]. ET_B_ receptor-mediated cerebral artery contraction curves were obviously enhanced in SHR compared with WKY. However, cerebral arterial ET_B_ receptor mRNA and protein expression in SHR were not markedly increased. These results suggest that the amount of ET_B_ receptor may not change, whereas receptor susceptibility increases. Contrarily, in SHR coronary arteries, ET_B_ receptor-mediated contraction, mRNA and protein expression levels were not significantly changed, suggesting that ET_B_ receptor amounts and activity in coronary arteries were unaltered.

However, controversy exists about the ET-induced vascular response in hypertension, depending on the animal hypertension model, hypertension duration, the experimental conditions used, and the blood vessels studied [40]. Tschudi and Lüscher reported that SHR coronary artery contractile responses to ET-1 were decreased compared with WKY [40]. They determined that ET-1-induced tension was approximately 0.4 and 1.4 mN/mm in SHR and WKY, respectively. However, KCl-induced contraction also decreased. KCl-induced wall tension (0.28 ± 0.03 mN/mm) in SHR was approximately one-third of that in WKY (0.87 ± 0.05 mN/mm). ET-1 is a receptor-operated agonist, and KCl is a receptor-independent agonist. The relative contractions (as a percentage of the KCl-mediated contraction) indicated the agonist or receptor sensitivity. ET-1-induced contractions relative to KCl were 143% and 161% in WKY and SHR, respectively. In our study, ET-1-induced contractions relative to KCl in the coronary artery were 156±5.8% and 152±5.0% in WKY and SHR, respectively. The relative contractions in the two experiments were similar. However, there was an important difference in ET-1-induced receptor-mediated contraction in the two experiments. Because ET_B_ receptor desensitization was performed, only ET_A_ receptor-mediated coronary contraction occurred in our experiment. However, in their experiment, coronary contraction was mediated by both ET_A_ and ET_B_ receptors. In their experiment, the contraction reflected the ET-1 function, whereas in our experiment, the contraction reflected the function of ET_A_ receptors. Thus, the two studies reflected two different ET family profiles.

MAPKs represent a serine/threonine protein kinase family that mediates fundamental biological processes and cellular responses to external stress signals. Many pathophysiological processes activate the MAPK pathway [41,42]. Previous studies demonstrated MEK/ERK1/2 activation in subarachnoid hemorrhages and following cigarette smoke exposure [28,43]. Molecular studies have demonstrated that the MEK/ERK1/2 pathway is responsible for upregulation of some G-protein coupled receptors such as ET receptors [44,45]. Therefore, the intracellular signal transduction pathway may be involved in hypertension-mediated receptor expression alteration. We determined that ERK1/2 phosphorylation was elevated in SHR cerebral and coronary arteries compared with WKY. Thus, basal ERK1/2 pathway activity is higher during hypertension, which might contribute to not only enhanced ET receptor expression but also to elevated ET receptor affinity in cerebral and coronary arteries in SHR. However, in SHR aortas, p38 MAPK activation plays an important role in ET-1-induced vasoconstriction maintenance [46]. These data suggest that the mechanism may be organ-specific.

In conclusion, the present study demonstrated that cerebral arterial ET_A_ receptor was upregulated in SHR. ET_A_ receptor affinity was increased, whereas receptor number was not increased in SHR coronary arteries. In addition, ET_B_ receptor susceptibility was increased, but the receptor amount may be not changed in cerebral arteries. In contrast, ET_B_ receptor expression levels and activity in coronary arteries were not altered. ERK1/2 pathway activation may play a role in hypertension-associated receptor alterations. Understanding receptor alterations may advance our knowledge of hypertension-associated cardiovascular disease, provide new insight for future analysis of the mechanisms underlying hypertension, and suggest possibilities for novel treatments.

## References

[1] Zeng C, Villar VA, Eisner GM, Williams SM, Felder RA et al. (2008) G protein-coupled receptor kinase 4: role in blood pressure regulation. Hypertension 51: 1449-1455. doi:10.1161/HYPERTENSIONAHA.107.096487. PubMed: 18347232. 18347232 10.1161/HYPERTENSIONAHA.107.096487 PMC3722601

[2] Edvinsson LI, Povlsen GK (2011) Vascular plasticity in cerebrovascular disorders. J Cereb Blood Flow Metab 31: 1554-1571. doi:10.1038/jcbfm.2011.70. PubMed: 21559027. 21559027 10.1038/jcbfm.2011.70 PMC3137480

[3] Cline DM (2008) Epidemiology of hypertension. Ann Emerg Med 51: S3-S4. doi:10.1016/S0196-0644(08)00358-2. PubMed: 18191291. 18191291 10.1016/j.annemergmed.2007.11.003

[4] Psaty BM, Furberg CD, Kuller LH, Cushman M, Savage PJ et al. (2001) Association between blood pressure level and the risk of myocardial infarction, stroke, and total mortality: the cardiovascular health study. Arch Intern Med 161: 1183-1192. doi:10.1001/archinte.161.9.1183. PubMed: 11343441. 11343441 10.1001/archinte.161.9.1183

[5] Agapitov AV, Haynes WG (2002) Role of endothelin in cardiovascular disease. J Renin Angiotensin Aldosterone Syst 3: 1-15. doi:10.3317/jraas.2002.001. PubMed: 11984741. 11984741 10.3317/jraas.2002.001

[6] Yanagisawa M, Kurihara H, Kimura S, Tomobe Y, Kobayashi M et al. (1988) A novel potent vasoconstrictor peptide produced by vascular endothelial cells. Nature 332: 411-415. doi:10.1038/332411a0. PubMed: 2451132. 2451132 10.1038/332411a0

[7] Arai H, Hori S, Aramori I, Ohkubo H, Nakanishi S (1990) Cloning and expression of a cDNA encoding an endothelin receptor. Nature 348: 730-732. doi:10.1038/348730a0. PubMed: 2175396. 2175396 10.1038/348730a0

[8] Li J, Cao YX, Liu H, Xu CB (2007) Enhanced G-protein coupled receptors-mediated contraction and reduced endothelium-dependent relaxation in hypertension. Eur J Pharmacol 557: 186-194. doi:10.1016/j.ejphar.2006.11.057. PubMed: 17204265. 17204265 10.1016/j.ejphar.2006.11.057

[9] Khalil RA (2011) Modulators of the vascular endothelin receptor in blood pressure regulation and hypertension. Curr Mol Pharmacol 4: 176-186. doi:10.2174/1874467211104030176. PubMed: 21222646. 21222646 10.2174/1874467211104030176 PMC3134623

[10] Hynynen MM, Khalil RA (2006) The vascular endothelin system in hypertension--recent patents and discoveries. Recent Pat Cardiovasc Drugs Discov 1: 95-108. doi:10.2174/157489006775244263. PubMed: 17200683. 10.2174/157489006775244263 PMC1351106 17200683

[11] Schiffrin EL, Touyz RM (1998) Vascular biology of endothelin. J Cardiovasc Pharmacol 32 Suppl 3: S2-13. PubMed: 9883741. 9883741

[12] Schneider MP, Boesen EI, Pollock DM (2007) Contrasting actions of endothelin ET(A) and ET(B) receptors in cardiovascular disease. Annu Rev Pharmacol Toxicol 47: 731-759. doi:10.1146/annurev.pharmtox.47.120505.105134. PubMed: 17002597. 17002597 10.1146/annurev.pharmtox.47.120505.105134 PMC2825895

[13] Schiffrin EL (1995) Endothelin: potential role in hypertension and vascular hypertrophy. Hypertension 25: 1135-1143. doi:10.1161/01.HYP.25.6.1135. PubMed: 7768553. 7768553 10.1161/01.hyp.25.6.1135

[14] Deng LY, Schiffrin EL (1998) Endothelin-1 gene expression in blood vessels and kidney of spontaneously hypertensive rats (SHR), L-NAME-treated SHR, and renovascular hypertensive rats. J Cardiovasc Pharmacol 31 Suppl 1: S380-S383. doi:10.1097/00005344-199800001-00108. PubMed: 9595489. 9595489 10.1097/00005344-199800001-00108

[15] Hansen-Schwartz J, Hoel NL, Zhou M, Xu CB, Svendgaard NA et al. (2003) Subarachnoid hemorrhage enhances endothelin receptor expression and function in rat cerebral arteries. Neurosurgery 52: 1185-1194. 10.1227/01.NEU.0000058467.82442.64. PubMed: 12699564. 12699564

[16] Stenman E, Malmsjö M, Uddman E, Gidö G, Wieloch T et al. (2002) Cerebral ischemia upregulates vascular endothelin ET(B) receptors in rat. Stroke 33: 2311-2316. doi:10.1161/01.STR.0000028183.04277.32. PubMed: 12215604. 12215604 10.1161/01.str.0000028183.04277.32

[17] Wackenfors A, Emilson M, Ingemansson R, Hortobagyi T, Szok D et al. (2004) Ischemic heart disease induces upregulation of endothelin receptor mRNA in human coronary arteries. Eur J Pharmacol 484: 103-109. doi:10.1016/j.ejphar.2003.11.001. PubMed: 14729387. 14729387 10.1016/j.ejphar.2003.11.001

[18] de Groot MC, Illing B, Horn M, Urban B, Haase A et al. (1998) Endothelin-1 increases susceptibility of isolated rat hearts to ischemia/reperfusion injury by reducing coronary flow. J Mol Cell Cardiol 30: 2657-2668. doi:10.1006/jmcc.1998.0822. PubMed: 9990537. 9990537 10.1006/jmcc.1998.0822

[19] Bauer M, Wilkens H, Langer F, Schneider SO, Lausberg H et al. (2002) Selective upregulation of endothelin B receptor gene expression in severe pulmonary hypertension. Circulation 105: 1034-1036. doi:10.1161/hc0902.105719. PubMed: 11877350. 11877350 10.1161/hc0902.105719

[20] Li J, Cao YX, Liu Y, Xu CB (2012) Minimally modified LDL upregulates endothelin type B receptors in rat basilar artery. Microvasc Res 83: 178-184. doi:10.1016/j.mvr.2011.12.001. PubMed: 22198335. 22198335 10.1016/j.mvr.2011.12.001

[21] Jie L, Yong-Xiao C, Zu-Yi Y, Cang-Bao X (2012) Minimally modified LDL upregulates endothelin type B receptors in rat coronary artery via ERK1/2 MAPK and NF- κB pathways. Biochim Biophys Acta 1821: 582-589. doi:10.1016/j.bbalip.2011.12.001. PubMed: 22198514. 22198514 10.1016/j.bbalip.2011.12.001

[22] Massart PE, Hodeige DG, Van Mechelen H, Charlier AA, Ketelslegers JM et al. (1998) Angiotensin II and endothelin-1 receptor antagonists have cumulative hypotensive effects in canine Page hypertension. J Hypertens 16: 835-841. doi:10.1097/00004872-199816060-00015. PubMed: 9663924. 9663924 10.1097/00004872-199816060-00015

[23] Sun T, Liu R, Cao YX (2011) Vasorelaxant and antihypertensive effects of formononetin through endothelium-dependent and -independent mechanisms. Acta Pharmacol Sin 32: 1009-1018. doi:10.1038/aps.2011.51. PubMed: 21818108. 21818108 10.1038/aps.2011.51 PMC4002526

[24] Cao L, Zhang Y, Cao YX, Edvinsson L, Xu CB (2012) Secondhand smoke exposure causes bronchial hyperreactivity via transcriptionally upregulated endothelin and 5-hydroxytryptamine 2A receptors. PLOS ONE 7: e44170. doi:10.1371/journal.pone.0044170. PubMed: 22952915. 22952915 10.1371/journal.pone.0044170 PMC3428315

[25] Högestätt ED, Andersson KE, Edvinsson L (1983) Mechanical properties of rat cerebral arteries as studied by a sensitive device for recording of mechanical activity in isolated small blood vessels. Acta Physiol Scand 117: 49-61. doi:10.1111/j.1748-1716.1983.tb07178.x. PubMed: 6858705. 6858705 10.1111/j.1748-1716.1983.tb07178.x

[26] Meng LQ, Tang JW, Wang Y, Zhao JR, Shang MY et al. (2011) Astragaloside IV synergizes with ferulic acid to inhibit renal tubulointerstitial fibrosis in rats with obstructive nephropathy. Br J Pharmacol 162: 1805-1818. doi:10.1111/j.1476-5381.2011.01206.x. PubMed: 21232035. 21232035 10.1111/j.1476-5381.2011.01206.x PMC3081123

[27] Li J, Cao YX, Cao L, Liu Y, Xu CB (2008) Heat stress alters G-protein coupled receptor-mediated function and endothelium-dependent relaxation in rat mesenteric artery. Eur J Pharmacol 588: 280-285. doi:10.1016/j.ejphar.2008.04.038. PubMed: 18511037. 18511037 10.1016/j.ejphar.2008.04.038

[28] Cao L, Zhang Y, Cao YX, Edvinsson L, Xu CB (2012) Cigarette smoke upregulates rat coronary artery endothelin receptors in vivo. PLOS ONE 7: e33008. doi:10.1371/journal.pone.0033008. PubMed: 22412974. 22412974 10.1371/journal.pone.0033008 PMC3296776

[29] Ahnstedt H, Stenman E, Cao L, Henriksson M, Edvinsson L (2012) Cytokines and growth factors modify the upregulation of contractile endothelin ET(A) and ET(B) receptors in rat cerebral arteries after organ culture. Acta Physiol (Oxf) 205: 266-278. doi:10.1111/j.1748-1716.2011.02392.x. PubMed: 22145714. 22145714 10.1111/j.1748-1716.2011.02392.x

[30] Cao L, Xu CB, Zhang Y, Cao YX, Edvinsson L (2011) Secondhand smoke exposure induces Raf/ERK/MAPK-mediated upregulation of cerebrovascular endothelin ETA receptors. BMC Neurosci 12: 109. doi:10.1186/1471-2202-12-S1-P109. PubMed: 22044770. 22044770 10.1186/1471-2202-12-109 PMC3219602

[31] Zulliger MA, Kwak NT, Tsapikouni T, Stergiopulos N (2002) Effects of longitudinal stretch on VSM tone and distensibility of muscular conduit arteries. Am J Physiol Heart Circ Physiol 283: H2599-H2605. PubMed: 12388322. 12388322 10.1152/ajpheart.00298.2002

[32] Larivière R, Sventek P, Schiffrin EL (1995) Expression of endothelin-1 gene in blood vessels of adult spontaneously hypertensive rats. Life Sci 56: 1889-1896. doi:10.1016/0024-3205(95)00163-Z. PubMed: 7746097. 7746097 10.1016/0024-3205(95)00163-z

[33] Edvinsson L, Povlsen GK (2011) Late cerebral ischaemia after subarachnoid haemorrhage: is cerebrovascular receptor upregulation the mechanism behind? Acta Physiol (Oxf) 203: 209-224. doi:10.1111/j.1748-1716.2010.02227.x. PubMed: 21087418. 21087418 10.1111/j.1748-1716.2010.02227.x

[34] Rossi GP, Colonna S, Belloni AS, Savoia C, Albertin G et al. (2003) Altered regulation of endothelin A receptor subtype in the cerebral arterioles in response to a Japanese-style diet, in stroke-prone hypertensive rats. J Hypertens 21: 105-113. doi:10.1097/00004872-200301000-00020. PubMed: 12544442. 12544442 10.1097/00004872-200301000-00020

[35] Cao L, Xu CB, Zhang Y, Cao YX, Edvinsson L (2013) Secondhand cigarette smoke exposure causes upregulation of cerebrovascular 5-HT(1) (B) receptors via the Raf/ERK/MAPK pathway in rats. Acta Physiol (Oxf) 207: 183-193. doi:10.1111/j.1748-1716.2012.02478.x. PubMed: 22883081. 22883081 10.1111/j.1748-1716.2012.02478.x

[36] Adner M, Uddman E, Cardell LO, Edvinsson L (1998) Regional variation in appearance of vascular contractile endothelin-B receptors following organ culture. Cardiovasc Res 37: 254-262. doi:10.1016/S0008-6363(97)00206-X. PubMed: 9539881. 9539881 10.1016/s0008-6363(97)00206-x

[37] Schroeder AC, Imig JD, LeBlanc EA, Pham BT, Pollock DM et al. (2000) Endothelin-mediated calcium signaling in preglomerular smooth muscle cells. Hypertension 35: 280-286. doi:10.1161/01.HYP.35.1.280. PubMed: 10642311. 10642311 10.1161/01.hyp.35.1.280

[38] Tostes RC, Wilde DW, Bendhack LM, Webb RC (1997) Calcium handling by vascular myocytes in hypertension. Braz J Med Biol Res 30: 315-323. doi:10.1590/S0100-879X1997000300004. PubMed: 9246229. 9246229 10.1590/s0100-879x1997000300004

[39] Beg SS, Hansen-Schwartz JA, Vikman PJ, Xu CB, Edvinsson LI (2007) Protein kinase C inhibition prevents upregulation of vascular ET(B) and 5-HT(1B) receptors and reverses cerebral blood flow reduction after subarachnoid haemorrhage in rats. J Cereb Blood Flow Metab 27: 21-32. doi:10.1038/sj.jcbfm.9600313. PubMed: 16736053. 16736053 10.1038/sj.jcbfm.9600313

[40] Tschudi MR, Lüscher TF (1995) Age and hypertension differently affect coronary contractions to endothelin-1, serotonin, and angiotensins. Circulation 91: 2415-2422. doi:10.1161/01.CIR.91.9.2415. PubMed: 7729029. 7729029 10.1161/01.cir.91.9.2415

[41] Wu DC, Ye W, Che XM, Yang GY (2000) Activation of mitogen-activated protein kinases after permanent cerebral artery occlusion in mouse brain. J Cereb Blood Flow Metab 20: 1320-1330. doi:10.1097/00004647-200009000-00007. PubMed: 10994854. 10994854 10.1097/00004647-200009000-00007

[42] Pearson G, Robinson F, Beers Gibson T, Xu BE, Karandikar M et al. (2001) Mitogen-activated protein (MAP) kinase pathways: regulation and physiological functions. Endocr Rev 22: 153-183. doi:10.1210/er.22.2.153. PubMed: 11294822. 11294822 10.1210/edrv.22.2.0428

[43] Beg SA, Hansen-Schwartz JA, Vikman PJ, Xu CB, Edvinsson LI (2006) ERK1/2 inhibition attenuates cerebral blood flow reduction and abolishes ET(B) and 5-HT(1B) receptor upregulation after subarachnoid hemorrhage in rat. J Cereb Blood Flow Metab 26: 846-856. doi:10.1038/sj.jcbfm.9600236. PubMed: 16251886. 16251886 10.1038/sj.jcbfm.9600236

[44] Nilsson D, Wackenfors A, Gustafsson L, Ugander M, Ingemansson R et al. (2008) PKC and MAPK signalling pathways regulate vascular endothelin receptor expression. Eur J Pharmacol 580: 190-200. doi:10.1016/j.ejphar.2007.10.071. PubMed: 18031734. 18031734 10.1016/j.ejphar.2007.10.071

[45] Cao YX, Xu CB, Luo GG, Edvinsson L (2006) Up-regulation of alpha1A-adrenoceptors in rat mesenteric artery involves intracellular signal pathways. Basic Clin Pharmacol Toxicol 98: 61-67. doi:10.1111/j.1742-7843.2006.pto_240.x. PubMed: 16433893. 16433893 10.1111/j.1742-7843.2006.pto_240.x

[46] Kwon S, Fang LH, Kim B, Ha TS, Lee SJ et al. (2004) p38 Mitogen-activated protein kinase regulates vasoconstriction in spontaneously hypertensive rats. J Pharmacol Sci 95: 267-272. doi:10.1254/jphs.FPJ03091X. PubMed: 15215652. 15215652 10.1254/jphs.fpj03091x

